# New Formulations of Polysaccharide-Based Hydrogels for Drug Release and Tissue Engineering

**DOI:** 10.3390/gels1010003

**Published:** 2015-01-29

**Authors:** Francesca Camponeschi, Andrea Atrei, Giulia Rocchigiani, Lorenzo Mencuccini, Marianna Uva, Rolando Barbucci

**Affiliations:** 1 Department of Biotechnology, Chemistry and Pharmacy of University of Siena, Via Aldo Moro 2, 53100 Siena, Italy; E-Mails: camponeschi@unisi.it (F.C.); atrei@unisi.it (A.A.); giulia.rocchigiani@hotmail.it (G.R.); lorenzo.mencuccini@unisi.it (L.M.); marianna.uva@unisi.it (M.U.); 2 Interuniversity Research Centre for Advanced Medical Systems (C.R.I.S.M.A.), Viale Giacomo Matteotti 15/16, 53034 Colle di Val d’Elsa, Italy

**Keywords:** polysaccharide-based hydrogels, thixotropy, injectable hydrogels, tissue engineering, drug delivery, magnetic nanoparticles

## Abstract

Polysaccharide-based hydrogels are very promising materials for a wide range of medical applications, ranging from tissue engineering to controlled drug delivery for local therapy. The most interesting property of this class of materials is the ability to be injected without any alteration of their chemical, mechanical and biological properties, by taking advantage of their thixotropic behavior. It is possible to modulate the rheological and chemical-physical properties of polysaccharide hydrogels by varying the cross-linking agents and exploiting their thixotropic behavior. We present here an overview of our synthetic strategies and applications of innovative polysaccharide-based hydrogels: hyaluronan-based hydrogel and new derivatives of carboxymethylcellulose have been used as matrices in the field of tissue engineering; while guar gum-based hydrogel and hybrid magnetic hydrogels, have been used as promising systems for targeted controlled drug release. Moreover, a new class of materials, interpenetrating hydrogels (IPH), have been obtained by mixing various native thixotropic hydrogels.

## 1. Introduction

Hydrogels are materials constituted by physically interacting or chemically cross-linked hydrophilic polymer chains, capable of absorbing a high amount of water. Water molecules penetrate into the interstitial spaces of the three-dimensional polymer network, making the hydrogel similar to biological tissues. The amount of water absorbed depends on the porosity of the hydrogels and on the hydrophilicity of the polymer’s functional groups [1,2].

Referring to the interactions involved in the building up of the networks, two main classes of hydrogels can be distinguished: (I) physical hydrogels and (II) chemical hydrogels. In physical hydrogels, polymer chains are held together by molecular entanglement and/or ionic, hydrogen bonds and/or dipolar interactions. These non-covalent interactions create dishomogeneity and therefore physical hydrogels do not present structural repeatability [3,4,5]. On the contrary, chemical hydrogels consist mainly of a covalent cross-linked network, obtained by the chemical bonding of polymer chains via the addition of various cross-linking agents able to react with specific functional groups on the polymer chain [6,7,8].

In addition to covalent cross-linking, every chemical hydrogel also present physical interactions, due to the aggregation of hydrophobic cross-linking agents and/or parts of the polymer. This implies that a certain degree of dishomogeneity is also present in chemical hydrogels; however, unlike physical ones, their mechanical properties are highly reproducible, thus offering the possibility to modulate their cross-linking degree.

In both cases, the density of cross-links is crucial in determining both properties and applications of the gels, as it is responsible for the swelling behavior and therefore for the combined solid-like and liquid-like characteristics. For this reason, chemical hydrogels appear more attractive than physical hydrogels.

## 2. Polysaccharide-Based Hydrogels

**Figure 1:**
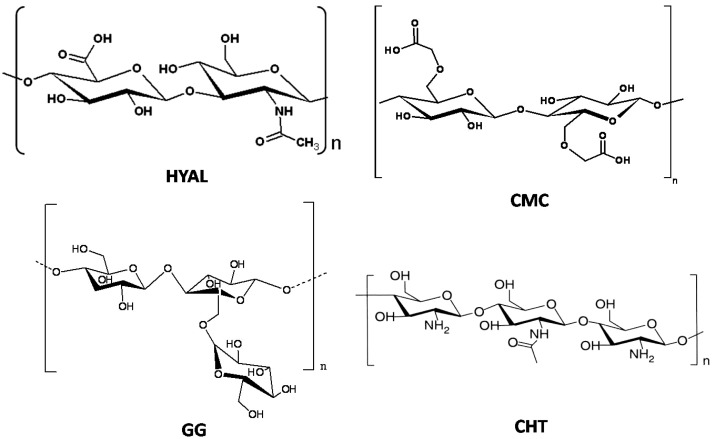
Chemical structure of the most popular polysaccharides used for the preparation of hydrogels. HYAL: hyaluronic acid; CMC: carboxymethylcellulose; GG: guar gum and CHT: chitosan.

Several classes of natural polymers can be used as starting materials to obtain chemical hydrogels. Among these, polysaccharides are one of the most useful because some of them have ionic groups, allowing an easy modification of their chemical properties by the introduction of new functional groups (e.g., amide, sulfate, phosphate and carboxylate groups) and also enzymes (e.g., superoxide dismutase). Some polysaccharides used for the preparation of hydrogels are shown in Figure 1: carboxymethylcellulose (CMC) [9,10], hyaluronic acid (HYAL) [11,12,13], guar gum (GG) and chitosan (CHT) [14,15,16,17,18]. At physiological pH CMC and HYAL molecules are negatively charged, while GG and CHT ones are, respectively, uncharged and positively charged. Thanks to the presence of several hydrophilic groups in the skeleton of these polysaccharides, hydrogels are able to uptake a large amount of water.

The chemically cross-linked polysaccharide-based hydrogels prepared in our laboratory show some peculiar features: (I) it is possible to control the cross-linking degree in a stoichiometric way and therefore the mechanical properties and the porosity of the hydrogel; (II) they are able to absorb a huge amount of water with respect to their weight (swelling capacity), which has a crucial role in determining the mechanical and chemical behavior of the hydrogels; (III) most of them are thixotropic, which makes these hydrogels injectable through a syringe, and therefore suitable for specific biomedical application.

### 2.1. Cross-Linking Density

The cross-linking degree is the first property that needs to be determined because it affects the mechanical and swelling properties of the hydrogels. Two methods have been successfully used in order to obtain such information: (I) potentiometric titrations, which allow the verification of the number of carboxylate groups (in CMC and HYAL-based hydrogels) or amine groups (in CHT-based hydrogels) of the polysaccharide not involved in the cross-linking reaction, *i.e.*, remaining free in the network and which can be titred by OH^−^ or H^+^, and (II) ^13^C NMR spectroscopy, by comparing the areas of specific NMR signals belonging to the polymer functional groups in the free or cross-linked form [19].

### 2.2. Swelling Behavior

The water uptake of the hydrogel is mainly affected by the hydrophilic functional groups of the polymer chains which interact in different ways with the water molecules [2,11].

Water is the main component of a hydrogel and can be schematically classified as (I) free water, in the outermost layer, which can be easily removed under mild conditions; (II) interstitial water, which is physically trapped in between the polymer chains; (III) bound water, which is directly bound to the polymer chains via the interactions with functional groups or ions. Being an integral part of the hydrogel structure, the bound water molecules can hardly be removed even at high temperature [10]. During the swelling process, the polar groups of the polymer chains are quickly hydrated by the first water molecules entering the hydrogel matrix (bound water): the network stretches out and the hydrophobic groups undergo aggregation. Afterwards, the network absorbs a further amount of water due to osmotic pressure (interstitial water and free water). The elastic force generated by covalent and physical cross-links opposes to the swelling process until reaching an equilibrium condition. The swelling degree of hydrogels give a measure of the uptake of water molecules. It can be calculated estimating the weight (Equation (1)) or height (Equation (2)) variation for each hydrogel, from the dry to the swollen state [17,18]:

% *S*_w_ = (*m_t_* − *m*_d_)/*m*_d_ × 100 (1)

% *S*_h_ = (*h*_s_ − *h*_0_)/*h*_0_ × 100 (2)

where *S*_w_ and *S*_h_ are the percentages of swelling at time *t*, *m_t_* and *h*_s_ are respectively the weight and the height of the swollen hydrogel at time *t*, *m*_d_ and *h*_0_ are respectively the weight and the height of the dry hydrogel.

**Figure 2:**
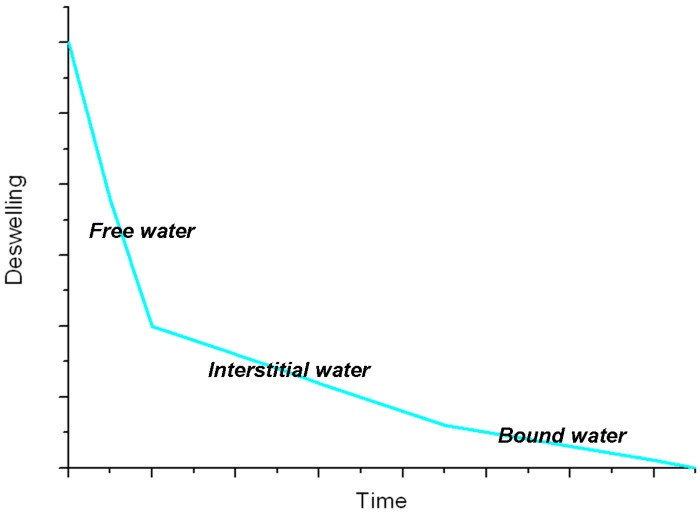
General trend of deswelling for a hydrogel [21].

The reverse process, *i.e.*, dehydration of the swollen hydrogel, is diffusion controlled and begins with the loss of free and interstitial water, involving a shrinking of the hydrogel. The following step is a further volume reduction due to the partial loss of the bound water, after which the macromolecular chains may organize to form amorphous and/or crystalline domains. The whole process can be monitored by plotting the deswelling factor [20] as a function of time (see Figure 2): from the slope of the curve we can discriminate among the different kinds of water molecules in the hydrogel [21]. However, a complete removal of bound water molecules never occurs. When the dry hydrogel is swollen in water again, the process does not produce the same arrangement of water molecules inside the polymeric network, leading to a different volume of the hydrogel due to different conformations, entanglements and physical interactions of the polymer chains.

### 2.3. Thixotropy

**Figure 3:**
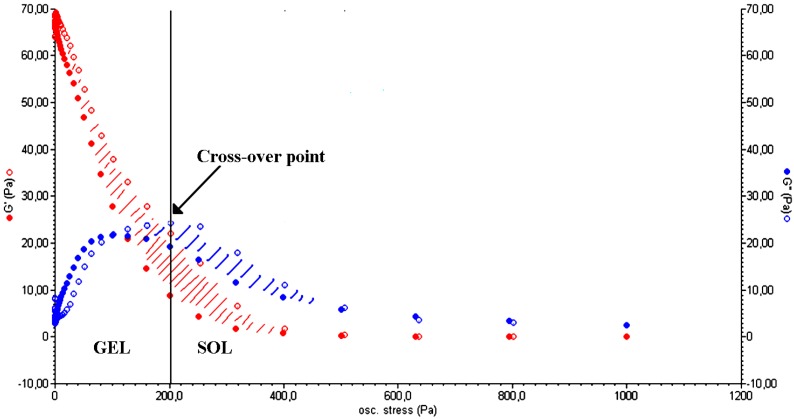
Elastic (G', **red**) and viscous (G", **blue**) moduli *vs.* oscillation stress for a CMC hydrogel in the stress sweep test. The cross-over point (G' = G") represents the point at which the sol–gel transition occurs (Adapted from reference [14]).

The capability of polysaccharide-based hydrogels to absorb a large amount of water makes them similar to highly viscous solutions and is the basis of the thixotropic behavior that characterizes some of them. Thixotropy is a property of some pseudo-plastic materials that allows them to fully recover their rheological properties (after a period of rest) following a progressive viscosity decrease due to a specific mechanical stress [22]. A hydrogel can be considered as several macro/micro-particles firmly connected to each other by water molecules. When an appropriate stress is applied (by a rheometer or by squeezing the hydrogel through a syringe), the particles slide on the water layer that connects them, generating a laminar flux. The system undergoes a reversible gel–sol isothermal transition. Once the stress is removed, the cross-linked structure slowly recovers as a consequence of the Brownian motion of particles [23] and the three-dimensional form of the material is restored*.* Figure 3 shows the typical trend of a stress sweep test performed on a polysaccharide-based hydrogel. For low values of oscillation stress, the storage modulus (G') is higher than the viscous one (G"), indicating the gel-like nature of the material. Over the cross-point (*i.e.*, the oscillation stress at which G' and G" have the same value) G" is higher than G', which is a characteristic behavior of a liquid. This implies a sol–gel transition of the material [24]. The thixotropic nature of the hydrogel is demonstrated by a double rheological graph, obtained by putting together an increasing and a decreasing shear curve. The two curves do not overlap, meaning that the material morphology has changed after the stress sweep test. The area within the two curves is called the hysteresis loop and it represents the energy dissipated in the sol–gel transition. Its presence confirms the thixotropic nature of a hydrogel [25].

The thixotropic behavior of the hydrogels can be explained considering the interaction of the polymer particles with water molecules. The mechanical properties of hydrogels are influenced by the presence of bound and semi-bound water molecules, which are able to make such materials tougher or softer [26]. Table 1 shows G' and G" values for some polysaccharide-based hydrogels in the native form and after they are forced to pass through a syringe needle. In the case of native hydrogels, strong polymer–water interactions occurring via hydrogen bonds are responsible for the higher values of G' and G" with respect to the squeezed hydrogel ones. Upon application of a shear stress, the strength of such physical interactions decreases, making the hydrogel softer. This behavior is probably due to a water molecule rearrangement inside the hydrogel. Since squeezed and native hydrogels show the same ability to absorb water, we can hypothesize that the rearrangement does not involve the free water molecules, but just bound and semi-bound water. In particular, water molecules undergo a transition from a bound state to a semi-bound state, inducing a decrease in the mechanical properties of the hydrogel.

**Table 1:** Storage modulus (G') and elastic modulus (G'') for polysaccharide hydrogels in native form and after being squeezed [14].

Hydrogel	State	G' (Pa)		G" (Pa)	
Carboxymethylcellulose	Native	550	±30	25	±1
	Squeezed	240	±20	20	±2
Hyaluronic acid	Native	970	±25	65	±10
	Squeezed	340	±20	45	±5
Chitosan	Native	4350	±650	165	±45
	Squeezed	3460	±150	230	±15

The infrared and nuclear magnetic resonance (NMR) spectra for a native hydrogel and for the same hydrogel once stressed do not show significant differences [27]. Therefore the passage through a syringe or the application of a stress during a rheological test induces changes in the hydrogel structure, but does not alter its chemical nature or affect its cross-linking degree [28].

## 3. Medical Applications of Injectable Polysaccharide Hydrogels

The well-known biocompatibility and the capability to be directly injected without altering the chemical structure are just two of the properties making polysaccharide-based hydrogels suitable materials for a wide range of medical applications that can be divided in two main fields:

- regenerative medicine;
- local therapy through drug delivery.

### 3.1. Minimally Invasive Surgery for Regenerative Medicine

Recently polysaccharide-based hydrogels became particularly attractive as matrices for the repair and regeneration of a wide variety of tissues and organs in the field of tissue engineering [29]. An ideal cell scaffold should provide an adequate mechanical support for cell growth, while ensuring the easy diffusion of nutrients and clearance of waste products [30,31]. Hydrogels are characterized by high permeability, which permits the exchange of oxygen, nutrients and water-soluble metabolites [32]. Here we report two applications of HYAL hydrogels in the tissue regeneration: cartilage and bone. Regenerative treatment of damaged cartilage is one of the fields where polysaccharide-based hydrogels have been used with more success. Hyaluronan, like other polysaccharides, is one of the most-used polymers for the treatment of osteoarthritis. HYAL plays a key role in the maintenance of the structural and functional characteristics of the joints and it determines the viscoelastic properties of the synovial fluid. In pathological processes, such as osteoarthritis, the molecular weight and concentration of hyaluronan in the synovial fluid can be reduced [33]. The result is a reduction in the viscoelasticity of the fluid and an increased susceptibility of cartilages to break down.

Hyaluronan-based hydrogels with a cross-linking degree of 50% (HYAL 50%) were used to improve the density and matrix appearance of cartilage in model animals [34]. It was observed that the surface of untreated lesions in the control group did not show any sign of repair in all samples and, after 50 days, defects were filled with irregular and very thin fibrous tissue. On the contrary, the local treatment with HYAL 50% improved the healing of damaged cartilage, which showed a smoother and more regular filling of surface lesions. Chondrocytes were present as cluster and columnar formations immersed in the hyaline-like matrix. At the same time, no evidence of tissue reaction or inflammation was observed and the permanence *in situ* of the hydrogel was longer than that of the HYAL polymer, thus giving the possibility to reduce the number of injections and, consequently, the risk of infection.

Nevertheless, in order to overcome the relatively high biodegradability rate of HYAL-based hydrogels, a new injectable derivative of CMC polymer, amidated carboxymethylcellulose (CMCA), was obtained through the insertion of amide groups into the CMC polymer backbone, with the aim of mimicking the biological activity in cartilage tissue repair that characterizes HYAL [34,35]. The results of both CMCA and HYAL hydrogels in the treatment of surgically created chondral defects in rabbit knees indicate that, after 50 days from the injection of the hydrogel, a layer of fibro-cartilaginous and hyaline-like tissue formed at the defect site.

**Figure 4:**
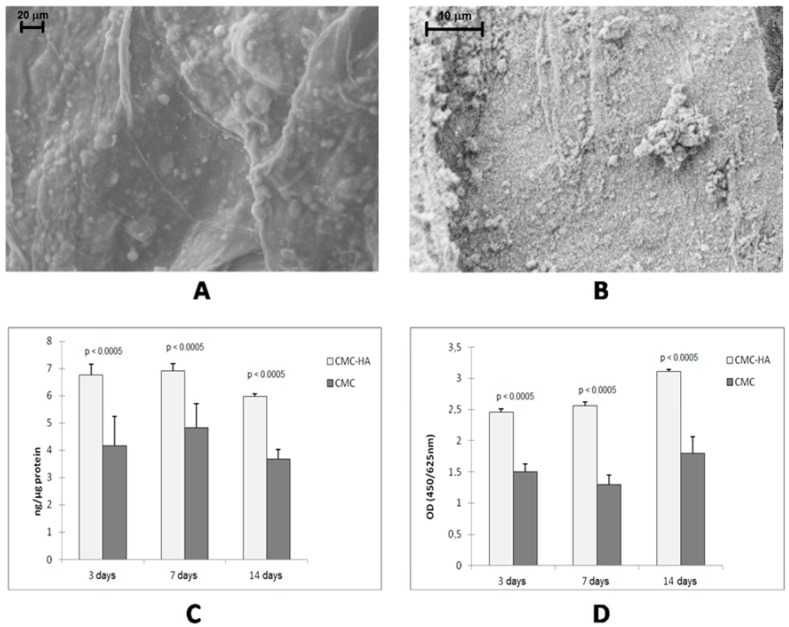
(**A**) FESEM image of hydrated CMC–HA and (**B**) high magnifications of HA crystals; (**C**) and (**D**) show MG63 osteoblast proliferation and activity markers after 3, 7 and 14 days of culture ((**C**) alkaline phosphatase (ALP) activity; (**D**) WST-1 assay) [48].

Bone tissue engineering is another field wherein hydrogels have been used with success. In particular, interesting results were obtained with the incorporation of inorganic components (such as hydroxyapatite (HA) nanoparticles, calcium phosphate or titanium dioxide micro- and nanoparticles) into specific bioactive hydrogels [36]. In fact, bone tissue consists of minerals and proteins [37,38]. In particular, 70% of the inorganic phase of bones is composed of collagen fibrils, made of HA nanocrystals linked to tropocollagen molecules. Therefore, micro- or nano-composite materials, containing synthetic HA, can be used to mimic natural bone. The presence of the inorganic phase gives to the matrix suitable mechanical properties and roughness which increase cell adhesion since osteoblast cells generally show enhanced proliferation and bioactivity on rough surfaces [39]. Several composites containing HA and bioactive polymers or proteins (such as collagen, chitosan, silk fibroin, gelatin and chondroitin sulfate) were prepared [40,41,42,43]. CMC-based hydrogels can be considered very good matrices thanks to the good biocompatibility, low toxicity and low degradation rate of CMC polymer in comparison to other polysaccharides [44,45]. The composite material can be obtained by co-precipitation of HA with CMC [46,47] or by incorporation of HA nanocrystals into the hydrogel matrix. The latter strategy allows a better control of the cross-linking degree and therefore of the mechanical properties of the hydrogel [48]. This strategy consists of swelling the freeze-dried hydrogel in an aqueous dispersion of HA nanocrystals. Ca^2+^ ions of the inorganic component and carboxylate groups of CMC interact to form the composite hybrid CMC-based hydrogel which shows the characteristics of both the components. In particular, the mechanical properties are improved while the water uptake capability is reduced with respect to pure CMC hydrogel. This behavior is due to the HA crystals which increase the consistency of the material and interact with the carboxylate groups of the polymer, leading to a decrease in the number of negatively charged groups on the hydrogel and thus to a reduction of the swelling degree in water. The presence of HA clusters on the surface of the CMC–HA hydrogel increases its surface roughness (Figure 4A,B), promoting the adhesion of osteoblast cells. By culturing osteoblast-like human cell lines in CMC-based hydrogel up to 14 days, a significant increase of cell differentiation (Figure 4C) and proliferation (Figure 4D) was observed for the CMC–HA hydrogel if compared to the pure CMC hydrogel [48].

### 3.2. Interpenetrating Hydrogels as Three-Dimensional Cell Scaffolds

The thixotropic nature and the resulting injectability of polysaccharide-based hydrogels offer a valid alternative to surgical implantation [17]. In the literature, mixed hydrogels are generally obtained by cross-linking two different polymers by chemical reactions [49,50]. The resulting hydrogel contains both polymers, but in percentages that depends on the number, type and reactivity of the chemical groups of the two different polymers. The characteristics and properties of mixed hydrogels are therefore not predictable. On the other hand, the design of a new class of injectable hydrogels with predictable properties can be obtained by using two different thixotropic hydrogels [17,32]. The two thixotropic hydrogels are squeezed through a syringe and then mixed in the fluid state. After a period of rest, the transformation from liquid to gel state occurs. In the resulting network, known as interpenetrating hydrogel (IPH), the polymer chains are held together by physical entanglement and/or non-covalent interactions. The IPH shows different mechanical, biological and physicochemical properties if compared to the native hydrogels. This strategy allows for prediction and modulation of the properties of the IPH, according to the type and relative percentage of each individual native component. Starting from CMC, CHT, GG and HYAL hydrogels, CMC–CHT, CMC–GG and CMC–HYAL IPHs were synthesized.

**Figure 5:**
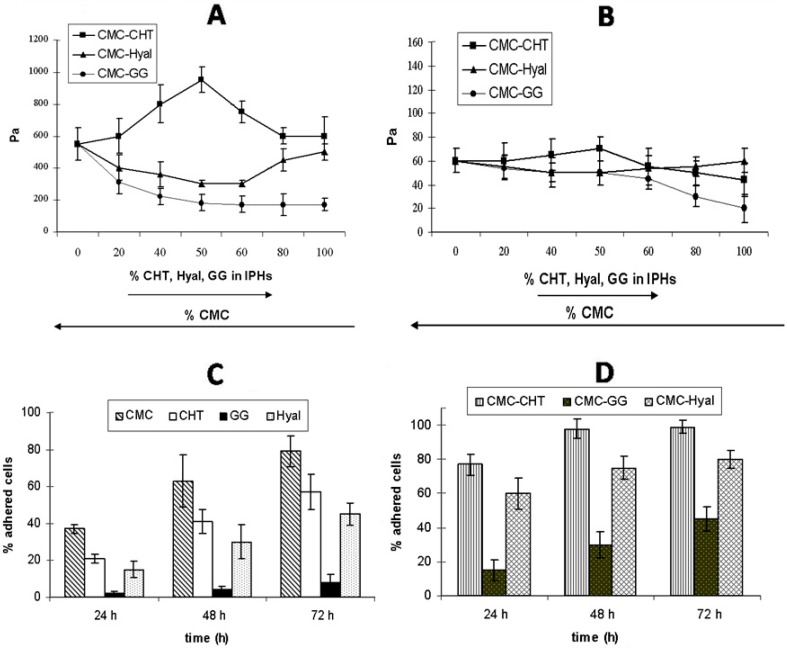
G' (**A**) and G" (**B**) values for mixed hydrogels, plotted as a function of the relative percentage of each native hydrogel in the IPHs; (**C**) and (**D**) show the percentage of fibroblasts *vs.* time (h) for each native hydrogel (**C**) and mixed hydrogel (**D**) [17].

The mechanical properties of the IPHs were different from those of the precursor hydrogels. For instance, in the case of the CHT–CMC hydrogels, G' values were higher than those of both the native hydrogels for all the percentage compositions. The increase in the mechanical properties can be explained by the emergence of interactions between the negatively charged CMC and positively charged CHT in the CMC–CHT IPH which may lead to a stiffer material. On the contrary, when hydrogels carrying the same charge (such as HYAL and CMC) were mixed, the G' values were always lower than those of the native compounds. Furthermore, the G' value of the CMC–GG IPH continuously decreased as the percentage of GG increased. This may be explained taking into account the absence of any interaction between CMC and GG. The trend of G" was consistent with that of G' for all the IPHs (see Figure 5A,B).

The biological performances of the IPHs containing 50% of each component were evaluated by seeding fibroblasts (NIH 3T3 cell line) on both the native hydrogels and IPHs. The growth trend of the NIH 3T3 cells cultured on the native hydrogels is reported in Figure 5C. The number of cells increased with time on all native hydrogels, demonstrating that cells are able to proliferate on these scaffolds. The number of cells increased with time on all the IPHs as well, and it was always higher than that of all the native hydrogels at each time-point (Figure 5D). The best results were obtained for CMC–CHT IPH (98% at 48 h), followed by CMC–HYAL (80% after 72 h) and CMC–GG (45% after 72 h). Cells cultured on the CMC–GG IPH showed the lowest cell proliferation, but the presence of CMC in the mixed hydrogel improved the biological performance of the material if compared to the GG hydrogel.

In conclusion, the interpenetrating method allows the formation of a mixed hydrogel *in situ* and offers the possibility to entrap cells in the bulk of the hydrogel when the transformation from liquid to gel occurs (Amber effect [51]).

### 3.3. Drug Delivery for Local Therapy

The ability of polysaccharide-based hydrogels to absorb large amounts of water makes them suitable to be used as injectable drug delivery systems for localized therapy. Hydrogels can be loaded with water-soluble drugs which interact with the polymer chains of the matrix via non-covalent interactions, allowing its release *in situ* under specific and controlled conditions.

One of the first applications of a polysaccharide-based hydrogel in this field concerns the use of a HYAL 50% hydrogel as a drug carrier for non-steroidal inflammatory drugs (NSAIDs), such as ibuprofen, for the treatment of knee osteoarthritis [52]. Ibuprofen has analgesic and anti-inflammatory properties by inhibiting articular chondrocyte apoptosis and dedifferentiation. However, systemic administration of NSAIDs has also adverse effects on the stomach, heart, and kidneys [53,54,55,56]. The local administration of NSAIDs through specific carriers such as hydrogels may represent a valid strategy to overcome such side effects.

In the case of HYAL 50% hydrogel loaded with ibuprofen–lysine, the observed therapeutic effects of HYAL 50% hydrogel on chondral lesions [34], as previously reported here, were maintained in the presence of ibuprofen–lysine.

Moreover, a bone mineral density (BMD) decrease was observed for model animals operated to legs and treated with HYAL 50% loaded with ibuprofen–lysine with respect to those treated with unloaded HYAL 50%. This may be considered an indirect parameter of leg usage and, consequently, of pain, since it is well known that decreased limb usage leads to a reduction of BMD [57,58].

The intrinsic drug-loading capability of polysaccharide-based hydrogels and their potential use as drug delivery systems can be combined with more sophisticated techniques in order to develop effective approaches for controlled drug release.

An example of this application is represented by electrochemotherapy (ECT), which is a tumor ablation technique based on the local application of short and intense electric pulses to tumor tissue [59]. This induces a variation in the transmembrane potential which leads to a reversible increase in the plasma membrane permeability via the formation of channels. Thanks to these channels, non-permeating drugs with high intrinsic cytotoxicity can be transported into cell interiors [60]. Thus, this technique can enhance chemotherapeutic drug activity directly at the tumoral site without affecting tissues not exposed to electric pulses [61].

The antibiotic bleomycin (BLM) is one of the most-used drugs in ECT. It cannot cross efficiently the plasma membrane [62], although electropermeabilization allows a certain number of BLM molecules to enter directly into the cytoplasm of the cell causing DNA fragmentations [63] and being therefore selectively cytotoxic for mitotic cells (*i.e.*, tumor cells). Unfortunately, ECT often involves not only tumor cells, but also stromal cells [64,65,66].

A guar gum (GG) hydrogel has been used as a bleomycin (whose molecule is positively charged) scaffold, in order to localize the drug only on the electropermeabilized tissue and to decrease its contact with non-treated tissues [67]. Since GG hydrogel is electrically neutral, it is inert to the current flow and at the same time allows the migration of the positively charged drug to the negative electrode. This migration can be targeted to the tumoral tissue by the continuous electric field and the release of BLM can be controlled since it occurs only during the application of a suitable electric field.

Time-of-flight secondary ion mass spectrometry (ToF-SIMS) analysis showed that the drug penetrates homogeneously into the polymer network and a very low amount of BLM was found at the surface [67]. The study showed that, in the absence of any external stimulus, the total amount of BLM released by GG hydrogel only reaches 41% after 100 min. On the contrary, when an electrical stimulus is applied, it is possible to obtain 100% of release after 12 pulses using a field strength of 200 V/cm or after 5 pulses with e field strength of 1000 V/cm.

The antitumor activity of the hydrogel-based system was tested *in vitro*. Different amounts of BLM (100, 50 and 30 μg) were used to treat cocultured tumoral NIH3T3 and endothelial HCAEC cells, showing that in the absence of an electric field BLM has a low effect on both cell lines. This is due to the high molecular weight of the drug that does not allow it to efficiently cross the plasma membrane [68]. On the contrary, when an electric field is applied to the adhered cells, a selective effect between primary and tumoral cells is observed, especially at an electric field strength of 600 V/cm.

The last application of polysaccharide-based hydrogels that will be considered here concerns magnetic hybrid hydrogels, which appear to be promising materials for targeted controlled drug release [69]. They consist of magnetic nanoparticles (NPs) embedded in a polysaccharide-based hydrogel matrix and show the peculiar properties of both the components [70].

Magnetic metal oxide NPs were used for the delivery of therapeutic molecules [71]. The drug delivery can be guided by directing the magnetic NP towards the target site in the body by means of a magnetic field gradient [72,73]. Once they have reached the target, the drug release from the NPs can be modulated by applying an alternating magnetic field. This should reduce the drug concentration at non-target sites, thus minimizing toxic side effects. Nevertheless, the main drawbacks of NPs are related to the small amount of drug that can be loaded on their surface, the possible deactivation of the drug occurring when it is bound to the NPs and the passive (uncontrolled) release of the drug (burst effect). Moreover, *in vivo* applications show that NPs can be attacked by the reticuloendothelial system (RES), which affects their half-life in the blood stream. Thus the injection of high concentrations of NPs for tumor treatment becomes mandatory, with potential systemic toxic effects. The incorporation of the metal oxide NPs into hydrogel matrices allows the overcoming of most of these problems. The main advantage offered by hydrogels is the capability of carrying a larger amount of drug if compared to dispersions of magnetic NPs. The presence of magnetic nanoparticles gives the hybrid system the ability to respond to external magnetic stimuli, such as static (SMF) and alternating magnetic fields (AMF), in order to move the hydrogel near the target site and then release the drug just at the appropriate moment [27,74,75,76,77]. NPs are embedded into hydrogels by loading them into the matrix during the swelling process or by simply mixing them with the preformed hydrogel during the gelation process [78,79,80,81,82].

These strategies share the limitation that NPs are only physically embedded within the hydrogel. This might imply a release of NPs from the hydrogel matrix into the external environment, with potentially toxic effects for the host tissue [83,84].

In order to overcome these problems, functionalized NPs can be used as cross-linking agents during the synthesis of hydrogels [27,85]. Similarly to other cross-linkers, the amount of NPs can be varied to modulate the physicochemical and mechanical properties of the resulting hydrogel. The presence of the polysaccharide in such a hybrid material makes it injectable near the target, minimizing drug wastage.

Several hybrid hydrogels sensitive to an external magnetic stimulus were obtained by using either CoFe_2_O_4_ or Fe_3_O_4_ NPs as cross-linking agents of CMC and HYAL polymer chains [27,86,87]. The general method relies on the introduction of NH_2_ groups on the NPs surface (NP–NH_2_) through the reaction with (3-aminopropyl)-trimethoxysilane (APTMS) [85].

This reaction leads to the formation of multiple layers of APTMS on the NP surface, as demonstrated by X-ray photoelectron spectroscopy studies carried out on CoFe_2_O_4_ and Fe_3_O_4_ NPs (an estimation for the thickness of APTMS films was obtained) [27].

The functionalized NP–NH_2_ can bind to the carboxylic groups of the CMC or HYAL polymer, allowing the formation of the relative hydrogels.

FT-IR analysis did not reveal any release of NPs in solution from the hybrid hydrogel, even after several washing cycles in water. The formation of covalent bonds between the NPs and the polymer chains therefore prevents the release of NPs [27].

The magnetic hybrid hydrogels show mechanical properties that are characteristic of polysaccharide-based chemical hydrogels, with storage modulus G' values (3000 and 350 Pa for CMC–NP and HYAL–NP, respectively) both higher than the loss modulus G" values (90 and 25 Pa for CMC–NP and HYAL–NP, respectively) [87]. Their rheological behavior is similar to that for hydrogels cross-linked with diamino–propane [17]. Moreover, the hybrid materials show G' values higher than G" even after squeezing through a syringe needle, confirming that they maintain gel-like characteristics. These measurements indicated the thixotropic nature of hybrid magnetic hydrogels.

The presence of magnetic NPs as cross-linkers in the hybrid hydrogel makes the system susceptible to the influence of external magnetic fields. The magnetic properties of CMC–NP hybrid hydrogels as carriers for the controlled release of a model drug (methylene blue, MB) have been investigated under the application of an AMF (4 Hz, 0.5 T) and a SMF (0.5 T) [88].

**Figure 6:**
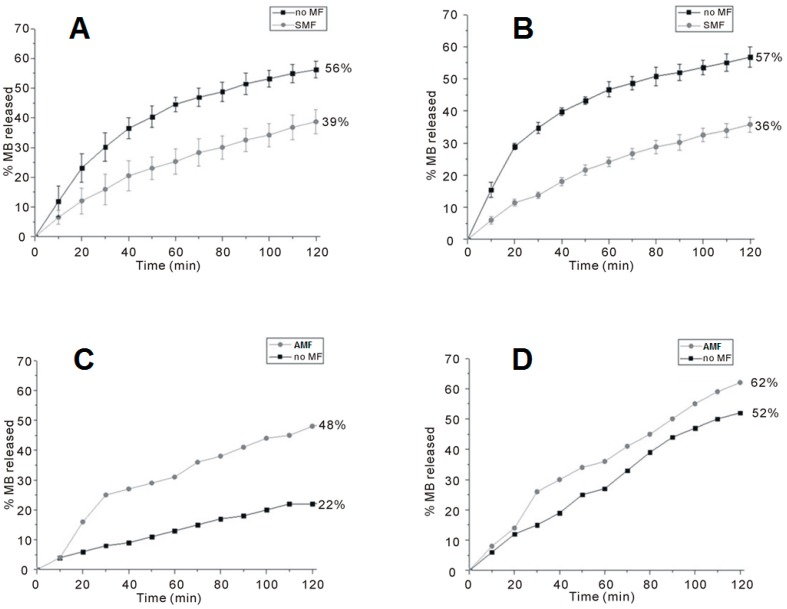
Release curve of MB from the magnetic hybrid hydrogels. (**A**) CMC–NP–50 in the absence (**black squares**) and in the presence (**grey circles**) of SMF; (**B**) CMC–NP–70 in the absence (**black squares**) and in the presence (**grey circles**) of SMF; (**C**) CMC–NP–50 in the absence (**black squares**) and in the presence (**grey circles**) of AMF (4 Hz, 0.5 T); and (**D**) CMC–NP–70 in the absence (**black squares**) and in the presence (**grey circles**) of AMF (4 Hz, 0.5 T) [88].

The release curves of two CMC-based hydrogels containing different amounts of CoFe_2_O_4_ NPs (50% and 70% relatively to the weight of the polymer, named CMC–NP–50 and CMC–NP–70, respectively) are shown in Figure 6. In both cases, under the SMF the samples release less MB than in the absence of any magnetic perturbation (Figure 6A,B). On the contrary, when subjected to a magnetic induction of 0.5 T at a frequency of 4 Hz, CMC–NP–50 and CMC–NP–70 show a greater MB release than without magnetic field (Figure 6C,D) [88].

**Figure 7:**
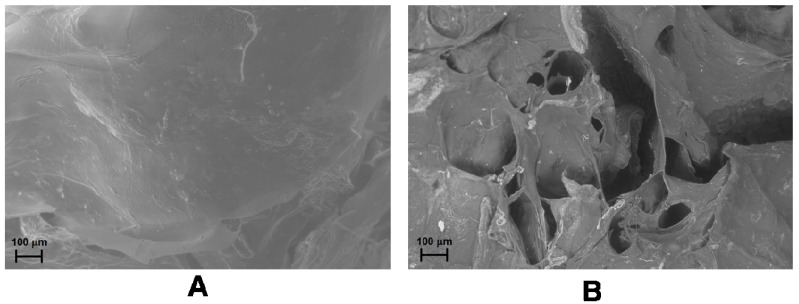
FE-SEM images of drug-loaded CMC–NPs hydrogel without application of magnetic field (**A**) and after the application of AMF (**B**) [88].

The release of any molecule from a magnetic hybrid hydrogel depends on the structural modifications occurring in the hydrogel as a consequence of the application of SMF or AMF. Field Emission Scanning Electron Microscopy (FE-SEM) analysis of the hydrogels shows a more packed structure with some rough protuberances when a SMF is applied to the hydrogel in comparison to the smooth surface of the native hydrogel. Moreover, the formation of several pores and unravelings on the hydrogel surface is observable after the application of AMF (Figure 7A,B) [88].

Therefore, SMF reduces the pore size in the hydrogel network, thus hindering the release of the model drug, while AMF increases the dimensions of the pores, allowing an easier release of the drug. A similar trend for the water uptake (WU) of the two hybrid hydrogels, measured under SMF and AMF, was observed. The WU value of both the hydrogels under AMF are in fact larger than in the absence of any magnetic field. The WU without the application of magnetic field is, in turn, greater than the value obtained under SMF.

**Figure 8:**
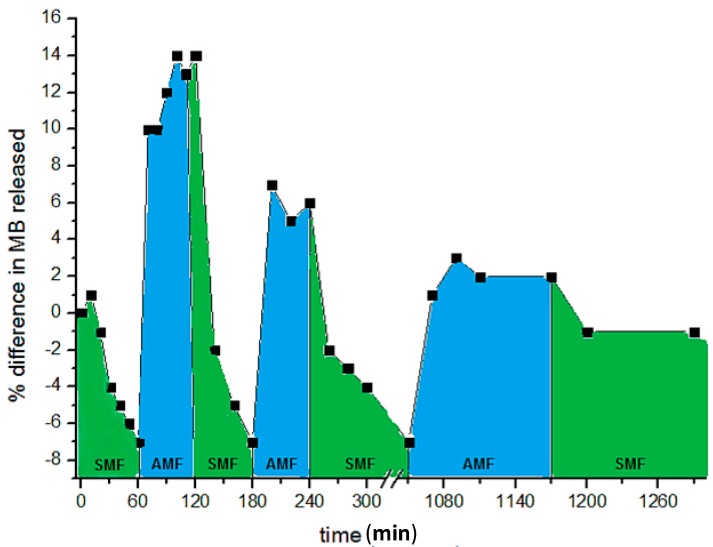
Release curve of methylene blue from CMC–NP–50 in 0.15 M NaCl at room temperature, under SMF (0.5 T) and AMF (0.5 T, 4 Hz) applied in sequence [88]. The NaCl solution was refreshed at every change of magnetic field.

By a cyclic sequence of AMF and SMF applications, it is possible to achieve a modulation of the drug release. Figure 8 shows the release trend of MB from CMC–NP–50 hydrogel under the application of SMF and AMF in sequence [88].

## 4. Conclusions

The results of this study will hopefully contribute to better understanding the behavior of chemically cross-linked polysaccharide networks. Polysaccharides are polymers derived from natural processes of growth and metabolism and offer many advantages over synthetic polymers, in particular their biocompatibility and similarity to the extracellular matrix.

A large group of polysaccharides consists of linear chains bearing several chemical groups in their backbone. Thus, the formation to hydrogels is quite easy and a small molecule can be used as cross-linkers to produce chemically cross-linked hydrogels. The presence of large amounts of water gives these materials a particular property: thixotropy, *i.e.*, hydrogels become liquid under mechanical stress or when squeezed through the needle of a syringe and then return to their gel state after a period of rest. This reversible sol–gel transition opens up several applications in regenerative medicine, for example in repairing cartilage or bone. Furthermore, by mixing two thixotropic hydrogels in the liquid state, new hydrogels, *i.e.*, the interpenetrating hydrogels, are obtained improving the biological and mechanical performances if compared to the native hydrogels. One of the most relevant applications of these polysaccharide-based hydrogels is the controlled drug release. The thixotropic properties of these materials allow them to be easily injected in the appropriate site of the body, preventing healthy tissues to get in contact with the drug.

Hybrid hydrogels with magnetic nanoparticles as cross-linkers can be targeted to diseased sites by means of magnets and the drug release can be remotely modulated by applying static and/or alternating magnetic fields.

Indeed polysaccharide-based hydrogels behave as smart materials and offer a variety of properties that can be exploited in several applications. Nevertheless, each polysaccharide exhibits properties and characteristics different from each other. A general behavior for all polysaccharide-based hydrogels does not exist and each hydrogel of a specific polysaccharide has to be individually studied.

Taking into account the extremely large number of natural polymers, this review is not exhaustive for the field of polysaccharide-based hydrogels and is meant to offer some examples and applications of these fascinating materials.
